# Electrostatic Map of the SARS-CoV-2 Virion Specifies Binding Sites of the Antiviral Cationic Photosensitizer

**DOI:** 10.3390/ijms23137304

**Published:** 2022-06-30

**Authors:** Vladimir Fedorov, Ekaterina Kholina, Sergei Khruschev, Ilya Kovalenko, Andrew Rubin, Marina Strakhovskaya

**Affiliations:** 1 Faculty of Biology, Lomonosov Moscow State University, 119234 Moscow, Russia; tenarra1@gmail.com (E.K.); styx@biophys.msu.ru (S.K.); ikovalenko78@gmail.com (I.K.); rubin@biophys.msu.ru (A.R.); marstr@biophys.msu.ru (M.S.); 2 Federal Scientific and Clinical Center of Specialized Types of Medical Care and Medical Technologies, Federal Medical and Biological Agency of Russia, 115682 Moscow, Russia

**Keywords:** SARS-CoV-2, photosensitizer, coarse-grain model, Brownian dynamics, electrostatic interactions

## Abstract

Electrostatics is an important part of virus life. Understanding the detailed distribution of charges over the surface of a virus is important to predict its interactions with host cells, antibodies, drugs, and different materials. Using a coarse-grained model of the entire viral envelope developed by D. Korkin and S.-J. Marrink’s scientific groups, we created an electrostatic map of the external surface of SARS-CoV-2 and found a highly heterogeneous distribution of the electrostatic potential field of the viral envelope. Numerous negative patches originate mainly from negatively charged lipid domains in the viral membrane and negatively charged areas on the “stalks” of the spike (S) proteins. Membrane (M) and envelope (E) proteins with the total positive charge tend to colocalize with the negatively charged lipids. In the E protein pentamer exposed to the outer surface, negatively charged glutamate residues and surrounding lipids form a negative electrostatic potential ring around the channel entrance. We simulated the interaction of the antiviral octacationic photosensitizer octakis(cholinyl)zinc phthalocyanine with the surface structures of the entire model virion using the Brownian dynamics computational method implemented in ProKSim software (version r661). All mentioned negatively charged envelope components attracted the photosensitizer molecules and are thus potential targets for reactive oxygen generated in photosensitized reactions.

## 1. Introduction

In the past two decades, the attention of researchers from different fields of science has been steadily drawn to coronaviruses (CoVs), enveloped viruses with single-stranded, positive-sense RNA genomes that infect birds and mammals [1,2]. CoVs are of particular concern due to their zoonotic potential and high plasticity, ability to infect multiple hosts, quick adaptation to new hosts and changing environmental conditions [3]. This is facilitated by large genomes, jumping mutations, and recombination potential.

In SARS-CoV-2, RNA encodes 16 non-structural proteins, nine accessory proteins, and four major structural proteins [4]—the nucleocapsid (N) protein, the envelope (E) protein, the membrane (M) protein, and the spike (S) protein. The N protein binds to viral RNA to form an RNP assembly [5] and determines its packaging in the ~80 nm-diameter lumen [6]. Through interactions with the M protein, N incorporates viral RNA in the budding virions independently of other proteins [7].

When CoVs bud in the endoplasmic-reticulum Golgi intermediate compartment (ERGIC), the nucleocapsid is covered by a lipid bilayer, derived from host lipids with embedded transmembrane viral proteins (E, M, and S) [8]. It is known that ER membranes are enriched in phosphatidylcholine (PC) and phosphatidylinositol (PI) [9], and, to mimic ERGIC, the lipid bilayer should contain PC, PI, phosphatidylethanolamine (PE), phosphatidylserine (PS), and cholesterol (Chol) [10]. SARS-CoV-2 and other CoVs use the small (76–109 amino acids) E protein to take control of ERGIC [11,12], but in the viral envelope, the E protein is represented by only a few copies. E is an integral membrane protein, with a single hydrophobic domain [12] that forms a homopentameric channel, with mild cation selectivity in negatively charged lipid membranes, termed “viraporin” [13].

The most abundant M protein is responsible for sorting and the incorporation of viral components in the budding virions and maintaining the shape and size of CoVs [4,7,14]. The M protein (about 230 amino acids) has three transmembrane domains and usually exists in the form of functional dimers [14,15]. It forms the matrix of the envelope and interacts with all other structural proteins.

The highly glycosylated S proteins (each about 1300 amino acids) form homotrimeric spikes, which protrude for about 20 nm from the viral membrane and make up the “corona” appearance. Each virus particle of SARS-CoV-2 bears 26 ± 15 spikes [6]. Host cell proteases, such as furin, cleave the S protein into S1 and S2 subunits in the infected cell, and thus in some CoVs the S1 and S2 subunits are associated non-covalently. The receptor-binding domain (RBD), responsible for coronavirus binding to a host cell receptor, is located in the S1 subunit of the spike protein, while the fusion peptide is assigned to the S2 subunit. The S2 single transmembrane domain anchors the S protein in the envelope [16].

Biophysics and structural biology provide key information for drug design to fight pandemic viruses [17,18]. However, the presence of lipid bilayers creates difficulties for the complete reconstruction of enveloped viruses by traditional methods of structural biology [19]. The complexity of the viral supramolecular structures, and the interactions between viral components and active substances, can be successfully described using so-called “computational microscopy”, with a spatiotemporal resolution unmatched by other methods [20].

Among the advances in computational virology bringing us closer to creating realistic models of whole virions are: the study of the structural dynamics of viral capsids, by employing a coarse-graining (CG) molecular dynamics method [21]; atomic HIV-1 capsid model [22]; simulation of Herpes simplex virus type 2 B-capsid and chromatin models in GENeralized-Ensemble Simulation System (GENESIS) [23]; a dynamic and integrative computational model of an influenza A virion [24]; computational model of the envelope of the dengue virion [25]; Martini CG models of intact SARS-CoV and SARS-CoV-2 envelopes [26]; and multiscale CG models of the SARS-CoV-2 virion [27,28].

In this study, we utilized the CG model of SARS-CoV-2 developed in [28], and kindly provided by W. Pezeshkian, D. Korkin and S.J. Marrink, to simulate the interaction of the photosensitizer (PS) octakis(cholinyl)zinc phthalocyanine (ZnPcChol^8+^) with the surface structures of the whole virion. We have recently shown that ZnPcChol^8+^ is a potent antiviral PS against bovine CoV [29], H5N8 avian influenza virus [30], and SARS-CoV-2 [31] in vitro. Proteins and unsaturated lipids of the viral envelope are readily oxidized with reactive oxygen species and free radicals generated by photoactivated PS in photodynamic reactions. As the photodynamic impact increased, the successive morphological changes in the H5N8 avian influenza virus and bovine CoV were as follows: the disappearance of the spikes, the change in the shape and size of the viral particles, the destruction of the envelope, and the complete collapse of the viral structure [29,30]. We analyzed electrostatic potential and simulated the binding of ZnPcChol^8+^ to the S protein of SARS-CoV, MERS-CoV, and SARS-CoV-2 [31,32] by means of Brownian dynamics (BD). In all three studied S proteins, we found a major binding site for ZnPcChol^8+^ at the connection of the S protein stalk and the head, adjacent to the heptad repeat 2 (HR2) domain. Hence, we created the electrostatic map of the whole SARS-CoV-2 virion and extended PS interaction simulations to the entire viral envelope to create a model which predicts PS binding sites and relevant targets of the reactive oxygen.

## 2. Results

### 2.1. Electrostatic Potential Distribution of the SARS-CoV-2 Envelope

**Figure 1:**
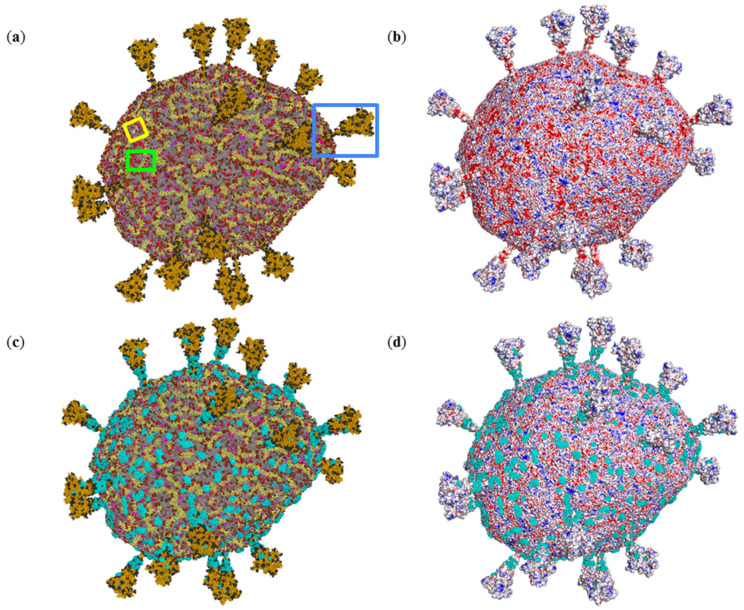
Three-dimensional structure of CG molecular model and electrostatic surface potential of SARS-CoV-2 virus envelope. CG beads colored in accordance with envelope component types (**a**). Rectangular boxes pick up the areas shown in Figure 2, Figure 3 and Figure 4 (green, yellow and blue, respectively). Surface of the virion colored by electrostatic potential value from −50 mV (red) to +50 mV (blue) (**b**). Electrostatic encounter complexes of ZnPcChol^8+^ with SARS-CoV-2 virion obtained by BD (**c**,**d**). The virion is colored in accordance with the envelope component types (**c**) and electrostatic potential (**d**). CG beads of neutral lipids, namely POPC, POPE and CHOL, are shown in gray; CG beads of negatively charged lipids are shown in shades of red: POPI molecules—in brown; POPS—in magenta; CDL2—in red. M proteins are shown in pale yellow: E proteins—in white; S proteins—in mustard with black glycans. ZnPcChol^8+^ molecules are visualized as cyan spheres.

The lipid and protein composition of SARS-CoV-2’s viral envelope is represented in Figure 1a. The CG model envelope includes three protein types, namely M, E, and S proteins. The M protein dominates and is presented in 1003 copies, the S protein is the only one protruding from the viral membrane and is not so numerous, it presented in 25 copies. In contrast to the other proteins, the viral membrane contains only two E proteins. The lipids of the viral membrane are presented by neutral palmitoyl oleoyl phosphatidylethanolamine (POPE, 20%), palmitoyl oleoyl phosphatidylcholine (POPC, 59%), cholesterol (CHOL, 4.5%) molecules and by negatively charged palmitoyl oleoyl phosphatidylinositol (POPI, 10%), palmitoyl oleoyl phosphatidylserine (POPS, 2%) and cardiolipin (CDL2, 4.5%).

**Figure 2:**
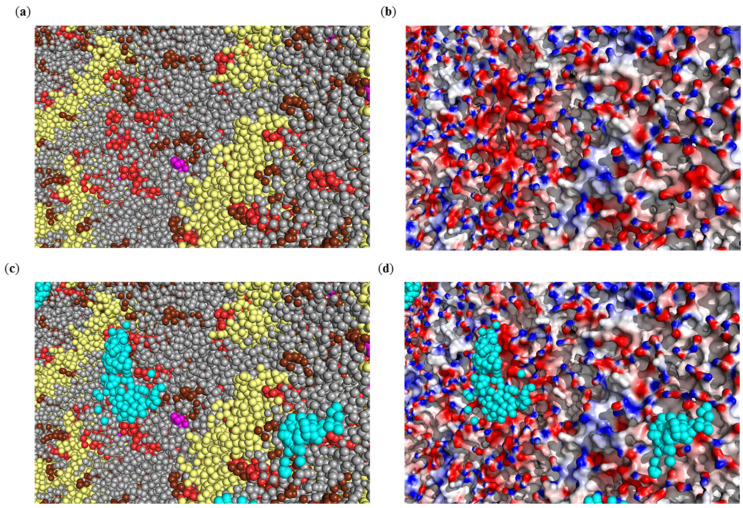
A fragment of the envelope of the SARS-CoV-2 virus, with M proteins and patches of charged lipids, which is an enlarged view of the rectangular area highlighted in green in Figure 1a (**a**). Molecular surface distribution of electrostatic potential from −100 mV (red) to +100 mV (blue) (**b**). Positions of the Zn atom in electrostatic encounter complexes of ZnPcChol^8+^ with SARS-CoV-2 virion obtained by BD (**c**,**d**). Virus envelope is colored in accordance with component types (**c**) and electrostatic potential (**d**). Neutral lipids, namely POPC, POPE, and CHOL, are shown as gray spheres; POPI molecules—as brown spheres; POPS—as magenta spheres; CDL2—as red spheres. Positions of the Zn atom of ZnPcChol^8+^ are shown as cyan spheres.

The SARS-CoV-2 virus envelope possesses a positive net charge of 8856 elementary charges, mainly due to the presence of more than a thousand positively charged M protein molecules (charge of each is +22). However, the SARS-CoV-2 virus envelope demonstrates a highly heterogeneous electrostatic potential field, with large alternating areas of positive and negative potential (Figure 1b). Negative patches of electrostatic potential are generated mainly by POPI (−1), POPS (−1), and CDL2 (−2) molecules and some negatively charged amino acids of S protein, as described in our previous papers [31,32]. Interestingly, negatively charged lipid molecules are prone to forming domains in the viral membrane (Figure 1 and Figure 2a), resulting in the existence of vast negatively charged areas on the surface of the SARS-CoV-2 virus envelope.

### 2.2. Interactions of Photosensitizer with the SARS-CoV-2 Envelope Revealed by Brownian Dynamics

To investigate which components of SARS-CoV-2 viral membrane attract ZnPcChol^8+^ molecule, we performed 40 thousand independent BD simulations of ZnPcChol^8+^ molecule relative to immobile viral envelope. Panels C and D of Figure 1 demonstrate the resulting 40,000 different electrostatic encounter complexes of photosensitizer with viral envelope, with electrostatic attraction energy exceeding 8 kT. Table 1 summarizes the statistics of contacts of the ZnPcChol^8+^ molecule with different components of the viral envelope.

About 43% of the ZnPcChol^8+^ molecules were found in electrostatic encounter complexes with SARS-CoV-2 membrane proteins. Among them, about 80% formed electrostatic contacts with S proteins; about 19%—with M proteins; and less than 1%—with E proteins. Most of the ZnPcChol^8+^ molecules bound to S proteins (91%) did not contact any membrane lipids or other proteins. On the contrary, almost all ZnPcChol^8+^ molecules bound to M proteins also interacted with negatively charged lipids (POPI, POPS, and CDL2); 70% of these molecules interacted with at least two charged lipid molecules. As for E proteins, about one-third of ZnPcChol^8+^ molecules bound to these proteins also formed contacts with negatively charged lipids, namely POPI (about 60%) and CDL2 (about 40%).

The remaining 57% of the ZnPcChol^8+^ molecules had no close contacts with membrane proteins but were bound to negatively charged lipids only; 63% of these molecules simultaneously interacted with at least two charged lipid molecules. Among the ZnPcChol^8+^ molecules interacting exclusively with charged lipid molecules (see Table 2), the most typical combination was the contact with one POPI molecule and one CDL2 molecule (19%). In 9% of cases, the ZnPcChol^8+^ molecule interacted with two POPI molecules, and in 7% of cases—with two CDL2 molecules. Another 7% of cases interacted with two CDL2 molecules and one POPI molecule.

From Table 2 it follows that in most cases, when ZnPcChol^8+^ interacts exclusively with lipids, the PS molecule forms contacts with several lipid molecules at once, due to the presence of patches of negatively charged lipids in the viral membrane. These patches are formed by either the same or different types of lipids (Figure 2a). The colocalization of negatively charged lipids POPI, POPS, and CDL2 results in formation of large regions of negative electrostatic potential (Figure 2b). As one can expect, these areas intensively attract ZnPcChol^8+^ molecules (Figure 2c,d).

On their own, the most abundant in the viral envelope are M proteins, with a total positive charge fail to attract PS molecules. However, M proteins due to their positive charge are colocalized with negatively charged lipids (Figure 2a), which attract ZnPcChol^8+^ molecules (Figure 2c). Therefore, in 19% of electrostatic complexes we observed contacts of PS with M proteins.

**Figure 3:**
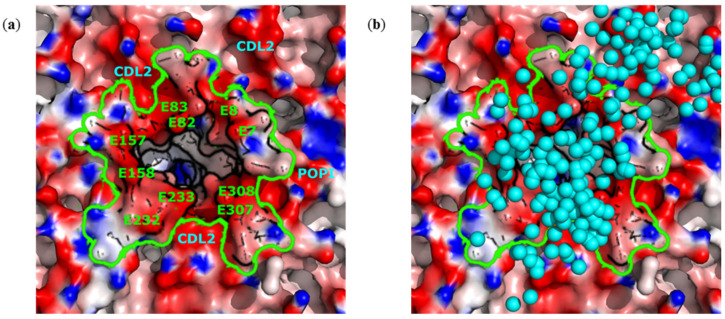
Detailed view of the surface distribution of electrostatic potential from −100 mV (red) to +100 mV (blue) of the pentameric E protein, embedded into the viral membrane (**a**), and its complexes with ZnPcChol^8+^ (**b**). CG beads representing the Zn atom of ZnPcChol^8+^ molecules in complexes with the SARS-CoV-2 envelope are shown as cyan spheres. Green line contours the borders of the E protein. Negatively charged surface amino acids of the E protein and negatively charged surrounding lipid molecules are labeled in panel (**a**). The view corresponds to the yellow frame in Figure 1a.

In the pentameric ion channel, each monomer, that is, the E protein, comprises three domains: the N-terminal, transmembrane and C-terminal domains. The N-terminal domain exposed outside the virion contains two negatively charged glutamate residues, E7 and E8. Despite the total positive charge (+10) of the E pentamer, these glutamates generate a ring area of negative electrostatic potential around the channel entrance (Figure 3a). In addition, the E protein is surrounded by negatively charged lipids, mainly CDL2, which further contribute to the negative surface potential around the channel. The resulting vast area of negative electrostatic potential intensively attracts ZnPcChol^8+^ molecules (Figure 3b).

**Figure 4:**
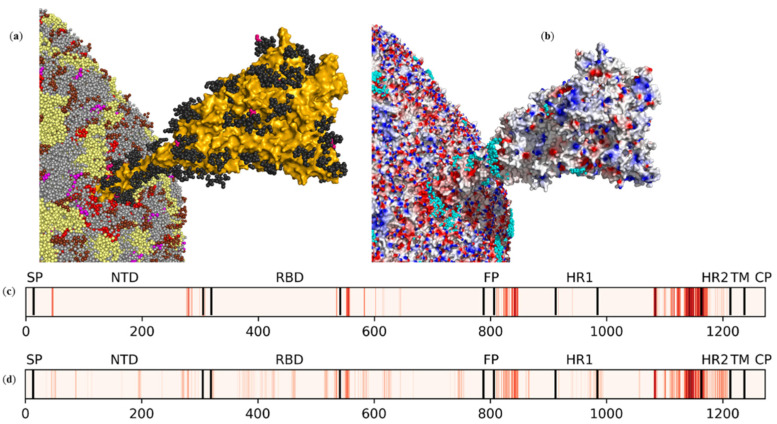
Effects of SARS-CoV-2 S protein glycosylation on ZnPcChol^8+^ binding. The local region of SARS-CoV-2 envelope with the S protein embedded into viral membrane (**a**), and molecular surface distribution of electrostatic potential from −100 mV (red) to +100 mV (blue) (**b**). Lipids and M proteins are the same as in Figure 1a,d. The S protein is shown as a mustard surface with black neutrally charged glycans and fuchsia negatively charged glycans. CG beads representing the Zn atom of ZnPcChol^8+^ molecules in complexes with SARS-CoV-2 envelope are shown as cyan spheres (**b**). The view corresponds to the blue frame in Figure 1a. The binding ability of non-glycosylated (**c**) and glycosylated (**d**) S proteins in relation to ZnPcChol^8+^.

Figure 4a demonstrates the section of the viral envelope with one of the 25 glycosylated S trimers embedded in the viral membrane. The S protein protruding from the viral membrane is one of the electrostatically attractive targets for ZnPcChol^8+^ [31,32]. As shown in these papers by means of all-atom molecular modeling, the electrostatic field is heterogeneously distributed on the surface of the “head” of the S protein. The “stalk” of the S protein is highly negatively charged and provides the major binding site to ZnPcChol^8+^ molecules (Figure 4c). In [31,32], we investigated the binding ability of non-glycosylated S protein. In the current CG model of SARS-CoV-2 envelope, the S protein trimer is glycosylated with 579 residues, among which 12 are negatively charged, that may influence ZnPcChol^8+^ binding. In accordance with our previous results, the major binding sites for ZnPcChol^8+^ are located in the linkers between FP and HR1, HR1 and HR2, as well as in the HR2 domain (Figure 4b,d). The presence of glycans on the surface of S proteins has little effect on ZnPcChol^8+^ binding. However, we found additional minor binding sites of ZnPcChol^8+^ on the surface of NTD and RBD domains of the S protein (Figure 4d).

## 3. Discussion

Electrostatic forces play a prominent role in the life cycle of coronaviruses, primarily by participating in the binding of the S protein to the host cell [33,34,35]. Mutations affecting the surface electrostatic potential may ultimately alter the infectivity, pathogenicity, and transmission rate of the virus [34].

In a polar aquatic environment, viruses have a pH-dependent surface charge due to the protonation/deprotonation of carboxyl and amino groups, and can be characterized by the so-called isoelectric point (IEP). IEP is considered a reliable quantitative characteristic that is useful in comparing the interactions of different viruses with the environment under different experimental conditions [36]. Michen and Graule [37] analyzed 152 IEP values from 104 viruses and found IEP to be in the range from 1.9 to 8.4, most often from 3.5 to 7, which may indicate an excess of acidic amino acids (aspartic acid and/or glutamic acid) exposed on the viral surface. The calculated IEP of SARS-CoV-2 ranges from 5.2 to 6.2, and when measured by means of chemical force microscopy in 20 mM salt is 5.2–5.3 [38]. The calculated IEP of the structural envelope S, E, and M proteins of SARS-CoV-2 are 6.24, 8.57, and 9.51, respectively [39]. In addition to proteins, phospholipids of the viral membrane, which originate from the membranes of the host cell, contribute to the total surface charge of enveloped viruses. Their diversity and variable ratio affect the total charge of virions, and complex lipid profiles cannot be predicted from the viral genome [36].

Another useful characteristic that reflects the total charge of a viral particle, its adsorption properties, and stability in dispersion is the zeta potential. The average zeta potential of the porcine coronavirus dispersed in double-distilled water was determined to be −25.675 mV [40]. However, zeta potential measurements require concentrated viral suspensions and are not applicable to intact pathogenic viruses.

To predict the precise binding of antiviral compounds, it is important to study the local electric charge distribution of the viral envelope components exposed to the surface. As we demonstrated, domains of S and M proteins protruding from the viral membrane have highly heterogeneous potential, while the exposed outside surface of the E protein is almost negatively charged. The total charge of proteins in the current model viral envelope is +21,261. The viral envelope is composed of a significant fraction of negatively charged lipids, contributing 12,405 elementary negative charges to the total +8856 net virion charge. As we demonstrated, negatively charged lipids form patches of condensed negative potential and provide more than a half of ZnPcChol^8+^ binding events. About 40% of ZnPcChol^8+^ binding events occur due to direct contact with envelope proteins, preferentially with S proteins. A high level of the S protein glycosylation almost does not influence the binding of polycationic PS. Glycan residues provide no steric barrier and leave enough space for PS binding. Additionally, predominantly neutral glycans do not shield the S protein surface charges, and so do not hinder electrostatic interactions with PS molecules.

The M protein with a total positive charge +22, the most abundant in the viral envelope, almost does not bind ZnPcChol^8+^ molecules. However, some binding events do happen due to the existence of local negative areas on the M protein surface. Despite the slight positive charge of the E protein, the N-terminal domain contains two glutamates, E7 and E8. These residues are exposed to the outside envelope surface and form a ring-like area of negative potential, contouring the entrance into the pentameric channel, attracting ZnPcChol^8+^ molecules.

We have recently shown that ZnPcChol^8+^ is a potent antiviral PS against bovine CoV [29], H5N8 avian influenza virus [30], and SARS-CoV-2 [31] in vitro. Using transmission electron microscopy with negative contrast, we have identified [29,30] a number of morphological changes common to the avian influenza virus and bovine coronavirus that occur during photodynamic inactivation: the disappearance of the spikes, the change in the shape and size of the viral particles, the destruction of the envelope, and the complete collapse of the viral structure. All of these lesions resulted in a loss of virus infectivity, which was determined by virus titration. In accordance with these morphological changes, we found that the most preferable binding site of PS is located on the S protein. Taking into account the small diffusion radius of singlet oxygen in the biological environment, the observed photosensitized damage to the viral membranes is in favor of close contacts of the PS with lipid membrane components. Actually, the negatively charged lipids in the viral model envelope provide multiple binding sites for PS molecules. Thus, the study of the detailed electrostatic map of the whole virion using a computer model provides unique opportunities to reveal the binding sites of charged molecules on the surface of the virus.

## 4. Materials and Methods

The interaction of PS with the SARS-CoV-2 envelope, both represented at the CG level, was studied by BD. The CG approach has the advantage of dealing with simplified molecular models, compared to the all-atom level. We used Martini CG models, based on the assumption that groups of several heavy atoms of particular chemical groups could be combined to represent one CG particle. The CG Martini force field proved to be an effective approach for studying different biomolecular systems [41].

In this study, we used the CG model based on Martini 3 force field of SARS-CoV-2 envelope developed in [28], and kindly provided to us by W. Pezeshkian, D. Korkin and S.-J. Marrink (8 February 2022 version). The lipids of the viral membrane were presented by 11817 POPE, 34860 POPC, 2658 CHOL, 5908 POPI, 1181 POPS, and 2658 CDL2 molecules. In total, 2 E proteins, 25 S proteins, and 1003 M proteins were embedded into the viral membrane. The CG model of ZnPcChol^8+^ was previously developed in our laboratory [42].

The electrostatic potential field and BD calculations were conducted using ProKSim (Protein Kinetics Simulator) software [43,44]. The Poisson–Boltzmann equation was used to calculate electrostatic potential field of SARS-CoV-2 virion and ZnPcChol^8+^ molecule. SARS-CoV-2 virion envelope was represented as a low dielectric area (ε = 2) with spatially fixed partial charges. The water solvent (dielectric constant ε = 80) with ions was described implicitly by the ionic strength 100 mM. Electrostatic cutoff radius was 3.5 nm.

In BD simulations, the SARS-CoV-2 virion was placed in the middle of a virtual reaction volume with mirror boundary conditions and dimensions of 200 × 200 × 200 nm. In each simulation, a ZnPcChol^8+^ molecule was randomly placed in the volume not occupied by the virion. The virion was immobile while the ZnPcChol^8+^ molecule was moving under the action of random and electrostatic forces. The calculation continued until the energy of electrostatic attraction of ZnPcChol^8+^ to the virion reached 8 kT. The final ZnPcChol^8+^ position with respect to the virion was saved for further analysis, and then the simulation was restarted from another random position of ZnPcChol^8+^. In total, we obtained 40,000 configurations of encounter complexes of ZnPcChol^8+^ with the SARS-CoV-2 virion.

We considered that contact between ZnPcChol^8+^ molecule and a specific component of the virion was formed if any CG bead of PS approached any CG bead of the virion within a distance of 0.6 nm. To characterize the interaction of PS with virion components throughout the obtained ensemble of structures, we used a custom Python script utilizing the MDAnalysis library [45], which identifies and counts particular components of viral envelope which are in contact with the PS molecule. Contact probability for particular amino acid residues of the S protein was calculated as the average fraction of contacts in PS electrostatic complexes with all 25 S protein molecules over the entire ensemble of structures. The contact probability for every amino acid residue was visualized on the S protein primary sequence by color. PyMOL software [46] was used to visualize molecular structures.

## 5. Conclusions

In conclusion, using a coarse-grained model of the SARS-CoV-2 envelope, we identified multiple heterogeneously distributed patches of negative potential at the envelope surface structures. These sites, formed mainly by negatively charged membrane phospholipids, as well as exposed exterior negatively charged amino acid residues on the S proteins, attract positively charged PS molecules and are consistent with the previously observed spike loss and membrane destruction, as a result of the photodynamic inactivation of the coronavirus with the same PS. The obtained results of model experiments explain how positively charged antivirals can bind to the coronavirus envelope, despite its net positive charge.

## Figures and Tables

**Table 1:** Statistics of contacts of ZnPcChol^8+^ molecule with different components of the viral envelope.

Components of the Viral Envelope			Fraction, %
proteins		S	33.9
		M	8.4
		E	0.6
lipids	negatively charged	POPI	43.9
		CDL2	40.6
		POPS	11.5
	uncharged	POPC	61.1
		POPE	39.4
		CHOL	0.5

**Table 2:** Distribution of electrostatic complexes of ZnPcChol^8+^ with negatively charged lipids by the number of contacts with different lipid types. The cases with occurrence less than 2% are omitted.

Number of Lipid Partners of ZnPcChol^8+^	POPI	POPS	CDL2	Fraction, %
1	0	0	1	18
1	0	1	0	3
1	1	0	0	13
2	0	1	1	3
2	0	0	2	7
2	2	0	0	9
2	1	1	0	4
2	1	0	1	19
3	1	1	1	3
3	1	0	2	3
3	2	1	0	2
3	2	0	1	7
3	3	0	0	3

## Data Availability

Not applicable.
